# Supplementary material for: Characterization of a Novel Endophytic Actinomycete, Streptomyces physcomitrii sp. nov., and Its Biocontrol Potential Against Ralstonia solanacearum on Tomato
Source: Microorganisms. 2020 Dec 18;8(12):2025. doi: 10.3390/microorganisms8122025 (PMC7765990; doi:10.3390/microorganisms8122025)

Supporting Information

Characterization of a Novel Endophytic Actinomycete, *Streptomyces physcomitrii* sp. nov., and Its Biocontrol Potential against *Ralstonia solanacearum* on Tomato

Xiaoxin Zhuang[1†], Congting Gao[1†], Chenghui Peng[1], Zhiyan Wang[2], Junwei Zhao[1], Yue Shen[1*], Chongxi Liu[1,2*]

[1]Key Laboratory of Agricultural Microbiology of Heilongjiang Province, Northeast Agricultural University, Harbin 150030, China

[2]State Key Laboratory of Phytochemistry and Plant Resources in West China, Kunming Institute of Botany, Chinese Academy of Sciences, Kunming 650201, China

*Correspondence: Yue Shen and Chongxi Liu

E-mail: shenyuelele@163.com, xizi-ok@163.com

[†]These authors have contributed equally to this work

Table S1. GenBank accession numbers of the sequences for the *Streptomyces* sequences used.

Strain	Type strain	Whole genome	*trp*B	*rpo*B	*gyr*B	*atp*D	*rec*A
S.physcomitrii	LD120^T	JAAWWP000000000	–	–	–	–	–
S. azureus	NRRL B-2655^T	CP007699	KT389149	KT388799	KT384829	KT384480	KT385177
S. levis	NRRL B-16370^T	–	KT389290	KT388941	KT384970	KT384621	KT385320
S. anandii	NRRL B-3590^T	–	MG881219	MG881217	FJ406188	MG881213	MG881215
S. fulvissimus	DSM 40593^T	CP005080	–	–	–	–	–
S. vietnamensis	GIM4.0001^T	CP010407	–	–	–	–	–
S. ambofaciens	ATCC 23877^T	CP012382	–	–	–	–	–
S. venezuelae	NRRL B-65442^T	CP018074	–	–	–	–	–
S. fungicidicus	TXX3120^T	CP023407	–	–	–	–	–
S. griseoviridis	NBRC 12874^T	CP034687	–	–	–	–	–
S. luridiscabiei	LMG 21390^T	–	EF055124	EF055069	EF054961	EF031272	EF055014
S. pluricolorescens	AS.4.236^T	–	EF661807	EF661786	EF661744	EF661723	EF661765
S. davawensis	JCM 4913^T	HE971709	–	–	–	–	–
S. labedae	KCTC 19961^T	–	JF423963	JF424010	JF424103	JF424197	JF424057
S. griseomycini	NRRL B-5421^T	–	KJ137095	KJ137078	KJ137044	KJ137027	KJ137061
S. lusitanus	NRRL B-5637^T	–	KJ196374	KJ196372	KJ196368	KJ196366	KJ196370
S. althioticus	NRRL B-3981^T	–	KT389129	KT388779	KT384809	KT384460	KT385157
S. atrovirens	NRRL B-16357^T	–	KT389142	KT388792	KT384822	KT384473	KT385170
S. baliensis	NRRL B-24754^T	–	FJ406352	FJ406277	FJ406185	FJ406129	FJ406241
S. bikiniensis	NRRL B-2690^T	–	KT389155	KT388805	KT384835	KT384486	KT385183
S. corchorusii	NRRL B-2904^T	–	KT389177	KT388827	KT384857	KT384508	KT385205
S. flavofungini	NRRL B-12307^T	–	KT389222	KJ996247	KT384902	KT384553	KT385251
S. gancidicus	NRRL B-1872^T	–	KT389230	KT388880	KT384910	KT384561	KT385259
S. indiaensis	NRRL B-24311^T	–	KT389270	KT388921	KT384950	KT384601	KT385300
S. pharetrae	NRRL B-24333^T	–	KT389353	KT389004	KT385033	KT384683	KT385384
S. pseudoechinosporeus	NRRL B-16931^T	–	KT389362	KT389014	KT385043	KT384693	KT385394
S. roseolus	NRRL B-5424^T	–	KT389376	KT389028	KT385057	KT384707	KT385408
S. roseoviolaceus	NRRL B-12177^T	–	KT389379	KT389031	KT385060	KT384710	KT385411
S. thermocarboxydus	NRRL B-24316^T	–	KT389404	KT389055	KT385084	KT389404	KT385437
S. werraensis	NBRC 13404^T	–	KT389431	KT389082	KT385110	KT384762	KT385464
S. zaomyceticus	NRRL B-2038^T	–	KT389440	KT389091	KT385119	KT384771	KT385473
S. scabiei	NRRL B-1515^T	–	KT601917	KT601874	KT601788	KT601745	KT601831
Kitasatospora setae	NRRL B-16185^T	JNWY00000000	–	–	–	–	–

Table S2. MLSA distance values for selected strains in this study.

Strains: 1, *Streptomyces physcomitrii* LD120^{T}; 2, *S. azureus* NRRL B-2655^{T}; 3, *S. levis* NRRL B-16370^{T}; 4, *S. anandii* NRRL B-3590^{T}; 5, *S. fulvissimus* DSM 40593^{T}; 6, *S. vietnamensis* GIM4.0001^{T}; 7, *S. ambofaciens* ATCC 23877^{T}; 8, *S. venezuelae* NRRL B-65442^{T}; 9, *S. fungicidicus* TXX3120^{T}; 10, *S. griseoviridis* NBRC 12874^{T}; 11, *S. luridiscabiei* LMG 21390^{T}; 12, *S. pluricolorescens* AS.4.236^{T}; 13, *S. davawensis* JCM 4913^{T}; 14, *S. labedae* KCTC 19961^{T}; 15, *S. griseomycini* NRRL B-5421^{T}; 16, *S. lusitanus* NRRL B-5637^{T}; 17, *S. althioticus* NRRL B-3981^{T}; 18, *S. atrovirens* NRRL B-16357^{T}; 19, *S. baliensis* NRRL B-24754^{T}; 20, *S. bikiniensis* NRRL B-2690^{T}; 21, *S. corchorusii* NRRL B-2904^{T}; 22, *S. flavofungini* NRRL B-12307^{T}; 23, *S. gancidicus* NRRL B-1872^{T}; 24, *S. indiaensis* NRRL B-24311^{T}; 25, *S. pharetrae* NRRL B-24333^{T}; 26, *S. pseudoechinosporeus* NRRL B-16931^{T}; 27, *S. roseolus* NRRL B-5424^{T}; 28, *S. roseoviolaceus* NRRL B-12177^{T}; 29, *S. thermocarboxydus* NRRL B-24316^{T}; 30, *S. werraensis* NBRC 13404^{T}; 31, *S. zaomyceticus* NRRL B-2038^{T}; 32, *S. scabiei* NRRL B-1515^{T}; 33, *Kitasatospora setae* NRRL B-16185^{T}

Strain	MLSA (Kimura 2-parameter) distance																															
	1	2	3	4	5	6	7	8	9	10	11	12	13	14	15	16	17	18	19	20	21	22	23	24	25	26	27	28	29	30	31	32	33
1	-																																
2	0.108	-																															
3	0.102	0.056	-																														
4	0.118	0.109	0.093	-																													
5	0.113	0.120	0.111	0.128	-																												
6	0.110	0.096	0.086	0.110	0.097	-																											
7	0.097	0.095	0.081	0.087	0.107	0.101	-																										
8	0.109	0.101	0.086	0.119	0.100	0.036	0.102	-																									
9	0.090	0.088	0.078	0.084	0.104	0.088	0.044	0.089	-																								
10	0.089	0.093	0.076	0.081	0.107	0.097	0.064	0.099	0.067	-																							
11	0.113	0.118	0.112	0.126	0.005	0.097	0.107	0.100	0.105	0.104	-																						
12	0.087	0.090	0.080	0.075	0.107	0.093	0.055	0.097	0.042	0.072	0.108	-																					
13	0.095	0.081	0.067	0.083	0.106	0.087	0.062	0.087	0.056	0.071	0.106	0.058	-																				
14	0.096	0.097	0.080	0.075	0.107	0.100	0.058	0.101	0.048	0.076	0.107	0.028	0.062	-																			
15	0.091	0.072	0.058	0.074	0.108	0.091	0.064	0.094	0.059	0.066	0.106	0.059	0.056	0.059	-																		
16	0.089	0.089	0.079	0.072	0.101	0.093	0.051	0.098	0.040	0.070	0.102	0.014	0.056	0.025	0.054	-																	
17	0.094	0.097	0.079	0.075	0.105	0.097	0.056	0.100	0.050	0.074	0.105	0.027	0.060	0.014	0.059	0.024	-																
18	0.087	0.087	0.078	0.083	0.105	0.092	0.054	0.095	0.031	0.069	0.105	0.039	0.054	0.045	0.060	0.038	0.044	-															
19	0.109	0.096	0.088	0.130	0.128	0.111	0.112	0.115	0.108	0.102	0.128	0.109	0.108	0.122	0.107	0.107	0.116	0.106	-														
20	0.104	0.107	0.088	0.106	0.100	0.058	0.092	0.055	0.091	0.089	0.101	0.091	0.089	0.099	0.081	0.090	0.098	0.089	0.117	-													
21	0.086	0.079	0.067	0.076	0.101	0.085	0.063	0.092	0.061	0.066	0.099	0.064	0.060	0.060	0.046	0.055	0.061	0.060	0.109	0.083	-												
22	0.115	0.125	0.112	0.122	0.087	0.107	0.107	0.108	0.108	0.110	0.086	0.116	0.102	0.115	0.111	0.111	0.115	0.111	0.121	0.115	0.102	-											
23	0.089	0.089	0.082	0.073	0.103	0.096	0.053	0.101	0.042	0.072	0.104	0.013	0.074	0.027	0.056	0.005	0.026	0.040	0.107	0.091	0.058	0.112											
24	0.096	0.057	0.038	0.099	0.115	0.085	0.082	0.088	0.077	0.079	0.114	0.079	0.066	0.085	0.061	0.077	0.083	0.079	0.095	0.090	0.074	0.108	0.080	-									
25	0.091	0.086	0.065	0.084	0.105	0.090	0.067	0.095	0.064	0.067	0.104	0.069	0.075	0.061	0.051	0.061	0.062	0.060	0.114	0.087	0.025	0.105	0.064	0.072	-								
26	0.093	0.104	0.096	0.106	0.125	0.111	0.089	0.110	0.076	0.085	0.124	0.080	0.101	0.090	0.079	0.082	0.088	0.077	0.101	0.110	0.085	0.110	0.083	0.101	0.087	-							
27	0.118	0.105	0.091	0.111	0.102	0.057	0.103	0.052	0.100	0.099	0.101	0.105	0.052	0.103	0.096	0.098	0.104	0.098	0.118	0.056	0.087	0.114	0.101	0.092	0.089	0.123	-						
28	0.093	0.070	0.064	0.091	0.113	0.090	0.072	0.090	0.060	0.073	0.113	0.067	0.058	0.075	0.055	0.067	0.075	0.061	0.078	0.093	0.066	0.108	0.069	0.063	0.069	0.063	0.106	-					
29	0.094	0.093	0.083	0.078	0.107	0.095	0.056	0.097	0.045	0.074	0.107	0.026	0.060	0.020	0.058	0.021	0.017	0.044	0.113	0.096	0.059	0.111	0.022	0.084	0.062	0.086	0.103	0.069	-				
30	0.094	0.092	0.081	0.080	0.107	0.094	0.055	0.099	0.046	0.072	0.106	0.029	0.081	0.018	0.059	0.021	0.018	0.043	0.117	0.098	0.056	0.113	0.024	0.083	0.058	0.087	0.099	0.075	0.017	-			
31	0.101	0.111	0.096	0.096	0.100	0.057	0.0.85	0.047	0.078	0.085	0.099	0.085	0.081	0.083	0.078	0.078	0.083	0.076	0.122	0.054	0.074	0.104	0.081	0.096	0.078	0.097	0.060	0.086	0.081	0.081	-		
32	0.090	0.092	0.082	0.081	0.107	0.096	0.055	0.098	0.043	0.070	0.107	0.027	0.059	0.018	0.057	0.020	0.020	0.041	0.144	0.096	0.057	0.113	0.022	0.080	0.060	0.085	0.099	0.072	0.016	0.016	0.079	-	
33	0.153	0.173	0.166	0.158	0.163	0.159	0.154	0.159	0.150	0.154	0.162	0.152	0.156	0.155	0.154	0.149	0.154	0.152	0.162	0.155	0.154	0.153	0.151	0.166	0.151	0.167	0.165	0.151	0.154	0.155	0.155	0.150	-

Table S3. Cultural characteristics of strain LD120^T, *S. azureus* NRRL B-2655^T and *S. anandii* NRRL B-3590^T.

Characteristic	LD120^T	*S. azureus* NRRL B-2655^T	*S. anandii* NRRL B-3590^T
**Growth on ISP 1:**			
Aerial mycelium	White	None	Light Gray
Substrate	Pale Yellow	Grayish Yellow	Grayish Yellow
**Growth on ISP 2:**			
Aerial mycelium	White	White	White
Substrate	Moderate Greenish Yellow	Strong Greenish Yellow	Deep Greenish Yellow
Diffusible pigment	Moderate Greenish Yellow	Strong Greenish Yellow	Deep Greenish Yellow
**Growth on ISP 3:**			
Aerial mycelium	White	Grayish Olive Green	Gray Greenish Yellow
Substrate	Pale Yellow	Light Greenish Yellow	Moderate Greenish Yellow
Diffusible pigment	None	None	Moderate Greenish Yellow
**Growth on ISP 4:**			
Aerial mycelium	White	Greenish Gray	Pale Greenish Yellow
Substrate	Moderate Greenish Yellow	Pale Yellow	Grayish Greenish Yellow
Diffusible pigment	Light Yellow	None	None
**Growth on ISP 5:**			
Aerial mycelium	White	Light Gray	Light Gray
Substrate	Light Yellow	Strong Greenish Yellow	Moderate Greenish Yellow
Diffusible pigment	Light Yellow	Strong Greenish Yellow	Moderate Greenish Yellow
**Growth on ISP 6:**			
Aerial mycelium	White	Light Gray	Pale Greenish Yellow
Substrate	Light Olive Brown	Grayish Yellow	Grayish Greenish Yellow
Diffusible pigment	None	Moderate Yellow	Moderate Yellow
**Growth on ISP 7:**			
Aerial mycelium	White	White	Light Gray
Substrate	Light Olive Brown	Grayish Reddish Brown	Moderate Greenish Yellow
Diffusible pigment	Moderate Greenish Yellow	Pale Yellow	Moderate Greenish Yellow
**Growth on CA:**			
Aerial mycelium	White	Light Greenish Gray	Yellowish White
Substrate	Pale Yellow	Pale Yellow	Moderate Greenish Yellow
Diffusible pigment	None	Pale Yellow	Moderate Greenish Yellow
**Growth on NA:**			
Aerial mycelium	White	White	White
Substrate	Pale Yellow	Pale Yellow	Yellowish White
Diffusible pigment	Yellowish White	None	None
**Growth on MBA:**			
Aerial mycelium	White	White	Pale Greenish Yellow
Substrate	Light Greenish Yellow	Pale Greenish Yellow	Moderate Greenish Yellow
Diffusible pigment	Light Greenish Yellow	Moderate Greenish Yellow	Moderate Greenish Yellow

Fig. S1. Maximum-likelihood tree showing the phylogenetic position of strain LD120^{T}and related taxa based on 16S rRNA gene sequences. Only bootstrap values above 50 % (percentage of 1000 replications) are indicated. *Kitasatospora setae* KCTC 9793^T(KM6054) was used as the outgroup. *Bar*, 0.005 nucleotide substitutions per site.

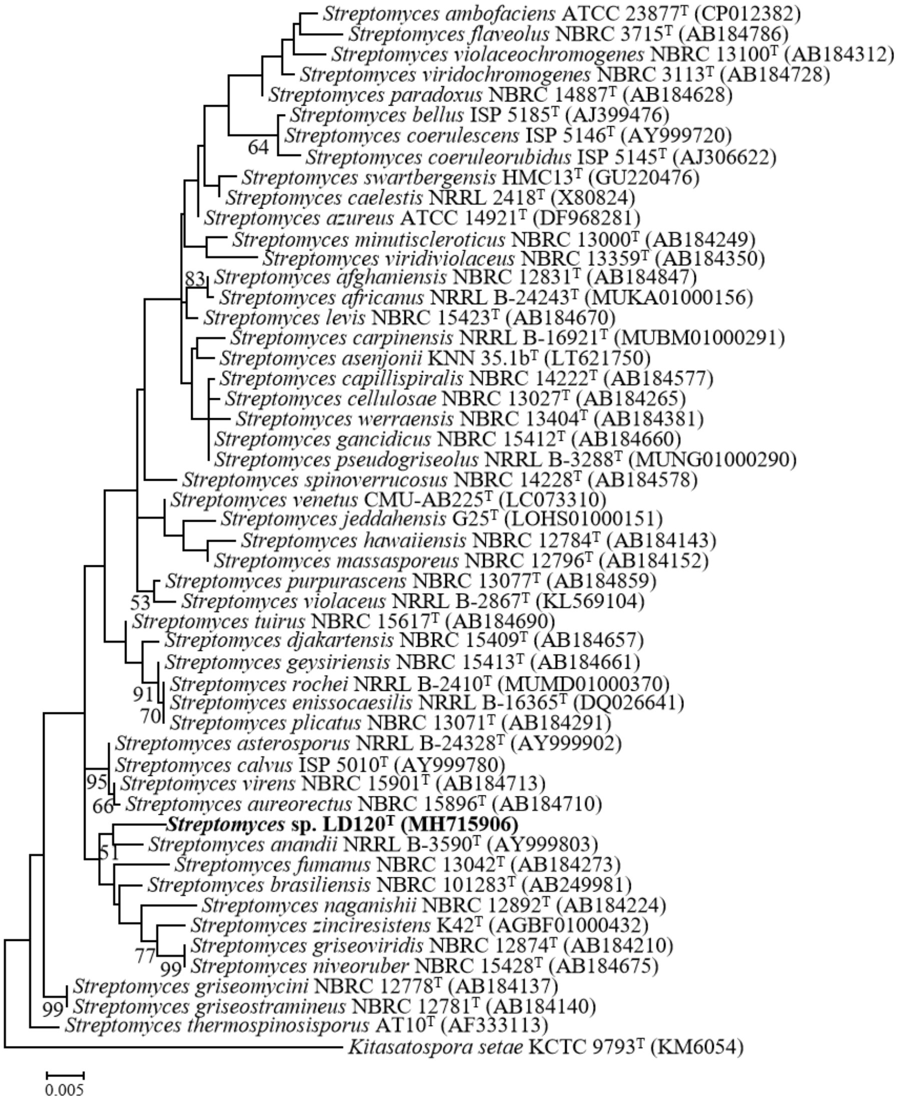

Fig. S2. Maximum-likelihood tree based on MLSA analysis of the concatenated partial sequences (2481 bp) from five housekeeping genes (*atp*D, *gyr*B, *rec*A, *rpo*B and *trp*B) of strain LD120^{T} and related taxa. Only bootstrap values above 50 % (percentages of 1000 replications) are indicated. *Kitasatospora setae* NRRL B-16185^{T} (KF591082) was used as the outgroup. *Bar*, 0.02 nucleotide substitutions per site.

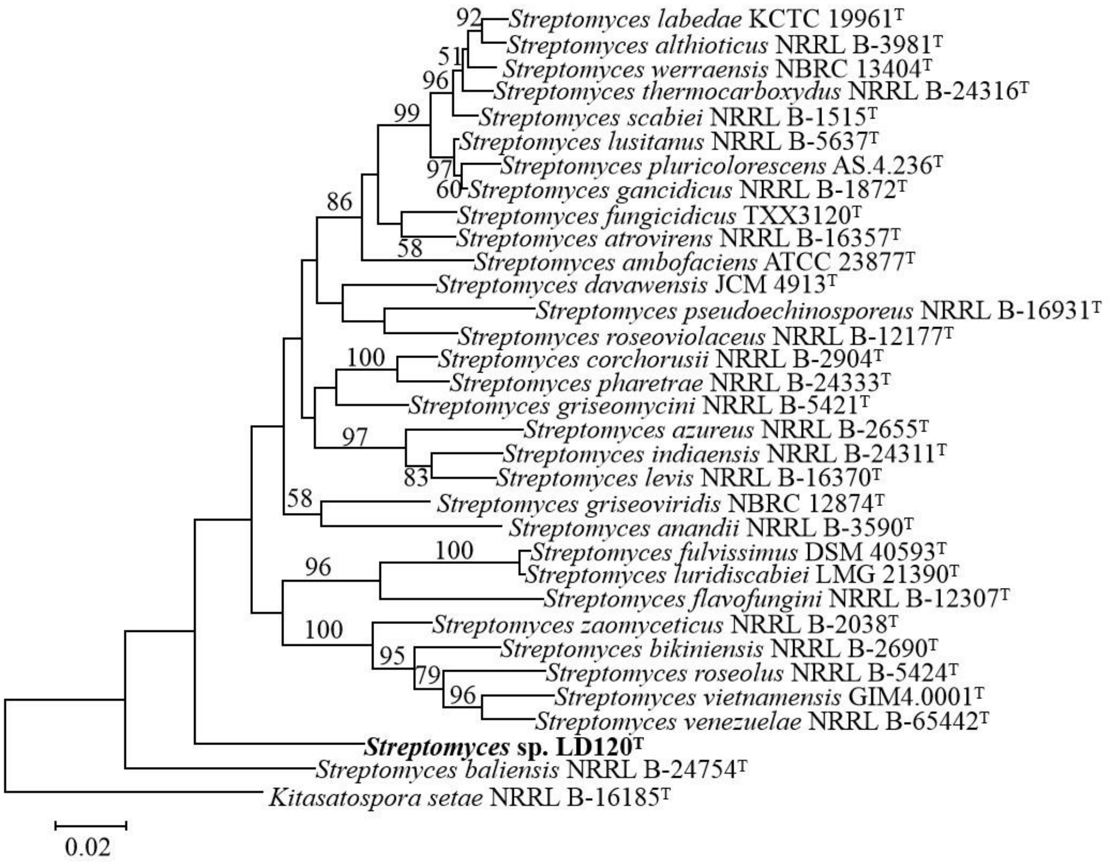

Figure S3. Whole-genome sequence tree generated with TYGS for strain LD120^T and validly published species of the genus *Streptomyces*. Tree inferred with FastME from GBDP distances calculated from genome sequences. Branch lengths are scaled in terms of GBDP distance formula d5; numbers above branches are GBDP pseudo-bootstrap support values from 100 repliacations.

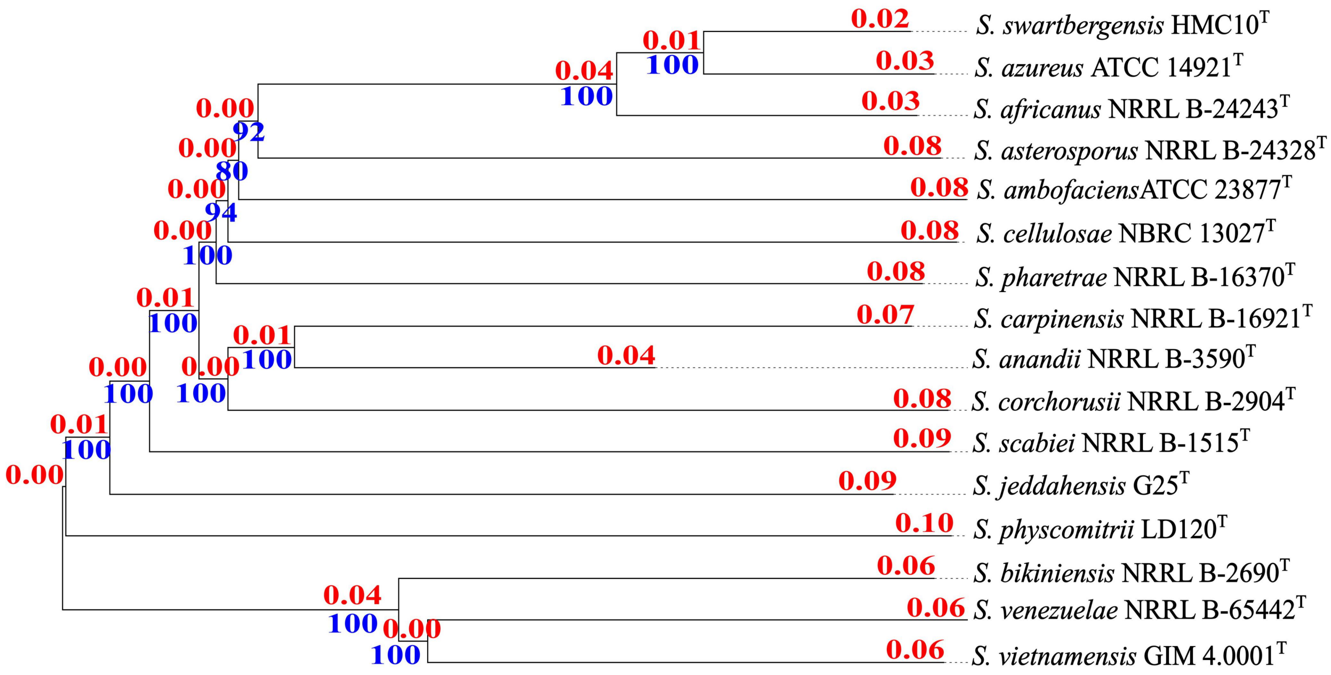

Fig. S4. Cultural characteristics of strains observed on ISP 1-7, CA, NA and MBA media at 28 ºC for 14 days.

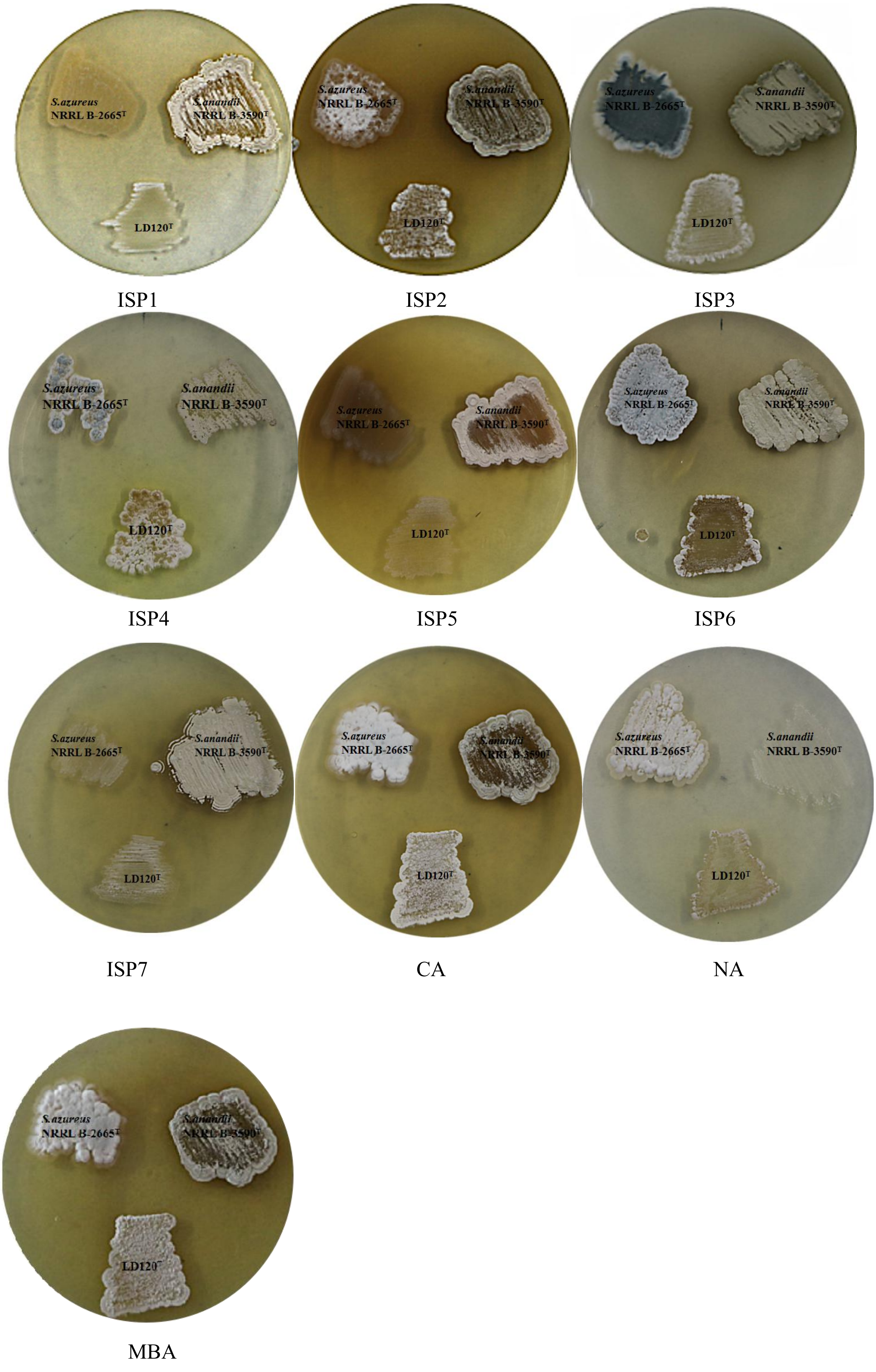

Fig. S5. The phospholipids of strain LD120^{T}. a, using molybdenum blue reagent; b, using ninhydrin reagent; c, using anisaldehyde reagent, d, using molybdophosphoric acid reagent. Abbreviations: DPG, diphosphatidylglycerol; PE, phosphatidylmethylethanolamine; PI, phosphatidylinositol.

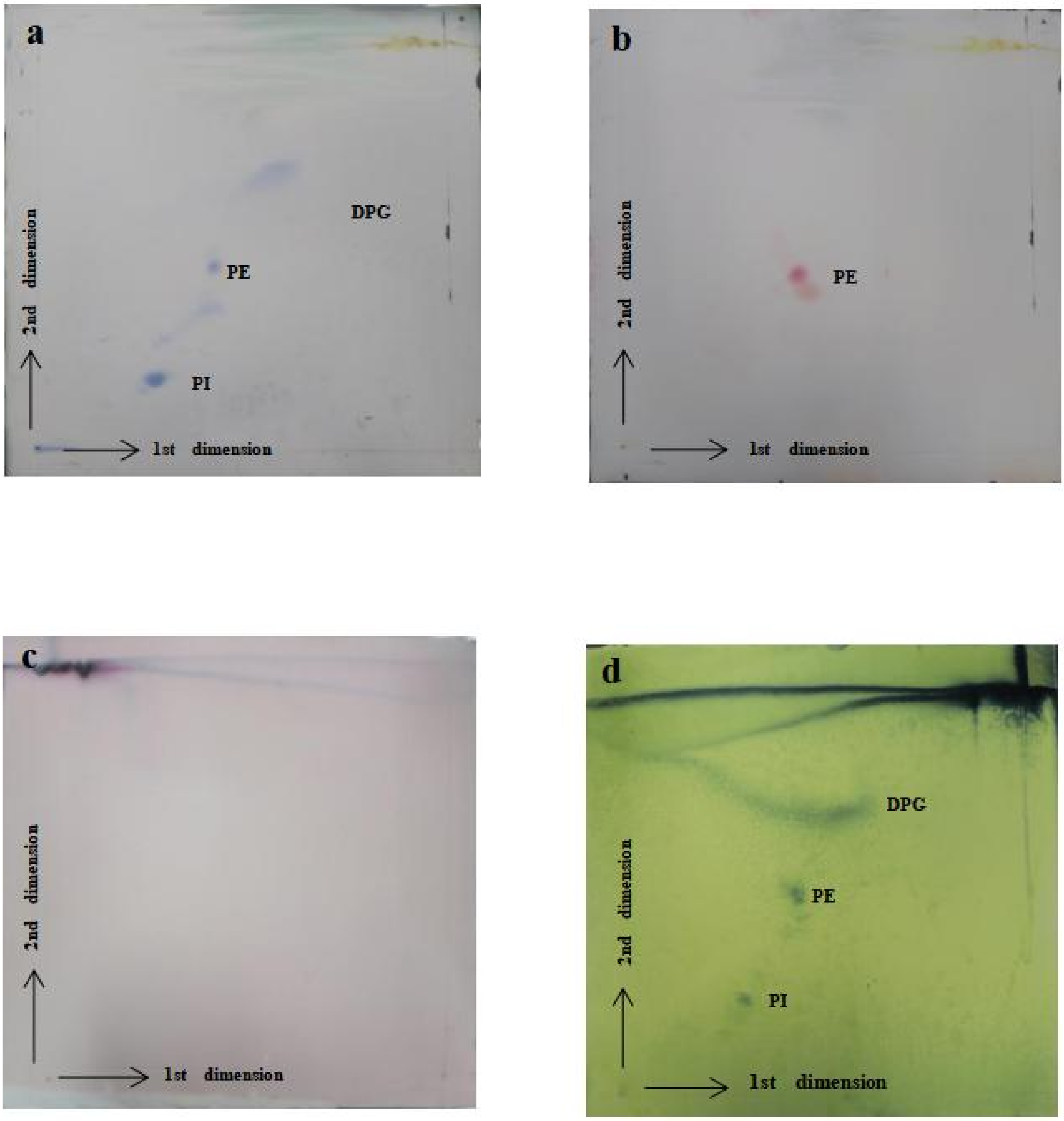

Supplement: Supplementary file 1 [file microorganisms-08-02025-s001.pdf]
